# Should individual timeline and serial CT/MRI panels of all patients be presented in acute brain insult cohorts? A pilot study of 45 patients with decompressive craniectomy after aneurysmal subarachnoid hemorrhage

**DOI:** 10.1007/s00701-022-05473-7

**Published:** 2023-01-30

**Authors:** Anniina H. Autio, Juho Paavola, Joona Tervonen, Maarit Lång, Terhi J. Huuskonen, Jukka Huttunen, Virve Kärkkäinen, Mikael von Und Zu Fraunberg, Antti E. Lindgren, Timo Koivisto, Jouni Kurola, Juha E. Jääskeläinen, Olli-Pekka Kämäräinen

**Affiliations:** 1 Neurosurgery, NeuroCenter, Kuopio University Hospital, PL 100, 70029 Kuopio, Finland; 2 Institute of Clinical Medicine, School of Medicine, Faculty of Health Sciences, University of Eastern Finland, Kuopio, Finland; 3 Neurointensive Care Unit, Kuopio University Hospital, Kuopio, Finland; 4 Department of Neurosurgery, Oulu University Hospital, Oulu, Finland; 5 Research Unit of Clinical Medicine, University of Oulu, Oulu, Finland; 6 Clinical Radiology, Kuopio University Hospital, Kuopio, Finland; 7 Center for Prehospital Emergency Care, Kuopio University Hospital, Kuopio, Finland

**Keywords:** Aneurysmal subarachnoid hemorrhage, EMS (emergency medical services), Neurointensive care, Individual timeline panels, Individual serial brain imaging panels, Brain tissue outcome

## Abstract

**Purpose:**

Our review of acute brain insult articles indicated that the patients’ individual (i) timeline panels with the defined time points since the emergency call and (ii) serial brain CT/MRI slice panels through the neurointensive care until death or final brain tissue outcome at 12 months or later are not presented.

**Methods:**

We retrospectively constructed such panels for the 45 aneurysmal subarachnoid hemorrhage (aSAH) patients with a secondary decompressive craniectomy (DC) after the acute admission to neurointensive care at Kuopio University Hospital (KUH) from a defined population from 2005 to 2018. The patients were indicated by numbers (1.–45.) in the pseudonymized panels, tables, results, and discussion. The timelines contained up to ten defined time points on a logarithmic time axis until death ($n=25$; 56%) or 3 years ($n=20$; 44%). The brain CT/MRI panels contained a representative slice from the following time points: SAH diagnosis, after aneurysm closure, after DC, at about 12 months (20 survivors).

**Results:**

The timelines indicated re-bleeds and allowed to compare the times elapsed between any two time points, in terms of workflow swiftness. The serial CT/MRI slices illustrated the presence and course of intracerebral hemorrhage (ICH), perihematomal edema, intraventricular hemorrhage (IVH), hydrocephalus, delayed brain injury, and, in the 20 (44%) survivors, the brain tissue outcome.

**Conclusions:**

The pseudonymized timeline panels and serial brain imaging panels, indicating the patients by numbers, allowed the presentation and comparison of individual clinical courses. An obvious application would be the quality control in acute or elective medicine for timely and equal access to clinical care.

## Introduction

Aneurysmal subarachnoid hemorrhage (aSAH), in most cases from a saccular intracranial aneurysm (sIA), is a complex and potentially critical systemic acute condition [83], requiring emergency medical service (EMS) care, immediate CT diagnosis, and transfer to the neurointensive care [5, 17, 22, 59, 97, 98, 104, 110, 112]. In aSAH, brain injuries may be caused by intracerebral hemorrhage (ICH) [15, 108], intraventricular hemorrhage (IVH) [7, 14], acute brain ischemia [53], acute hydrocephalus [49], increased intracranial pressure (ICP) [82, 114], herniations, spreading depolarization [19, 79], seizures [13], perihematomal edema [76], delayed brain injury [20, 64, 79], electrolyte disturbances, cardiopulmonary complications, central nervous system (CNS) or systemic infections, and complications of management.

Clinical articles on aSAH—and on other acute brain insults, including brain infarction, spontaneous ICH or IVH, and traumatic brain injury (TBI)—portray brain injuries and brain outcomes with words, phrases, numbers, scales, scores, risk ratios, areas under curves, tables and graphs, etc. We have difficulty in finding articles with *all patients’* individual (i) timeline panels and (ii) serial CT/MRI panels (brain outcome) from the ictus until one to three years: including recanalization in brain infarction; decompressive craniectomy (DC) in brain infarction, TBI, or aSAH; evacuation of ICH or IVH; “vasospasm” and delayed brain injury in aSAH. “Time is Brain” corresponds to “Time is Muscle” in acute coronary care [87]. In the quality control of acute brain infarct care, the term *workflow metrics* is used and *the key time points and periods* to the final recanalization are defined and recorded [29, 58, 92]. Actually, any area of Personalized Medicine would necessitate that the patient’s individual timelines are (i) monitored and recorded for the timely conduct of clinical care and (ii) compared to each other for the equal access to clinical services.

In this pilot study, we used DC as an indicator of escalating intracranial conditions since the aSAH ictus in the EMS and neurointensive care. We identified 45 DC patients admitted within 24 h from the CT diagnosis to the Kuopio University Hospital (KUH) Neurointensive Care Unit from 2005 to 2018. We compiled the individual (i) timeline panels (minute scale) since the EMS call and (ii) serial CT/MRI slice panels for the 25 deceased and for the 20 survivors.

Our aims were to illustrate in real life for the clinician readers to evaluate.

- the swiftness since the EMS call (112) and possible outliers during the EMS and KUH neurointensive care until the sIA occlusion,
- the sites and sizes of aneurysmal ICHs (aICHs) and aneurysmal IVHs (aIVHs),
- the development of brain edema, perihematomal edema, and ischemic brain injuries, and.
- the extent of brain injuries and brain atrophy at about 12 months after aSAH (*brain tissue outcome*) in the 20 survivors.

## Methods and materials

### KUH and EMS in Eastern Finland

**Fig. 1:**
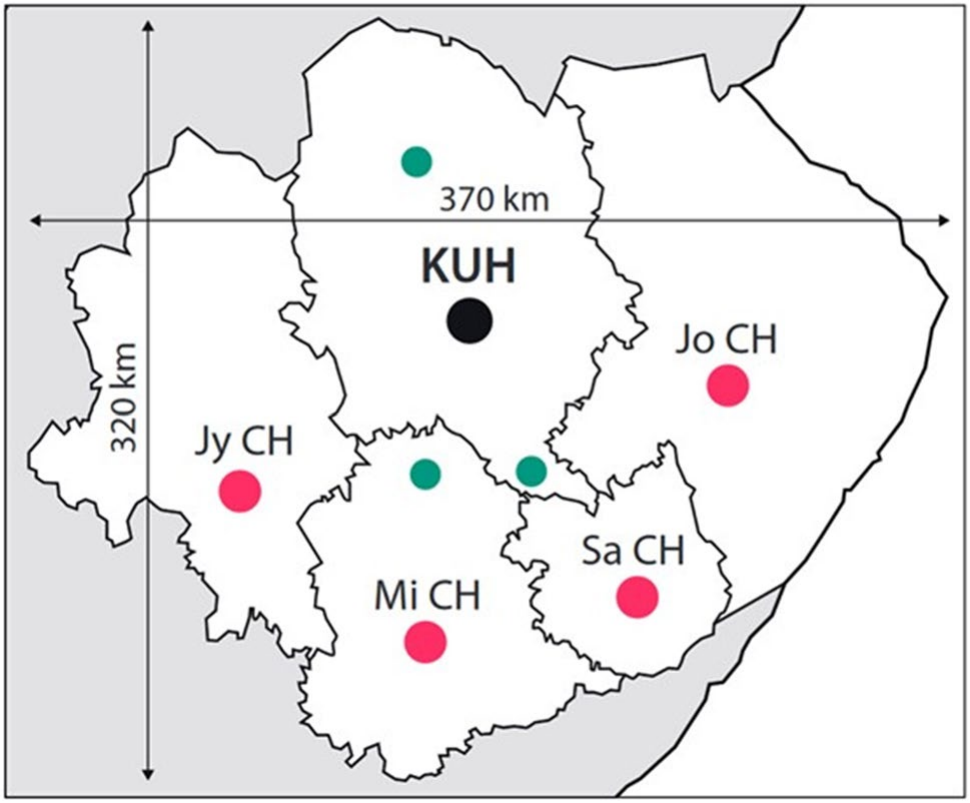
Map of Eastern Finland. The defined Eastern Finnish catchment population (805,133 in 2018) of the tertiary Kuopio University Hospital (KUH; black dot) is shown in white. The four referring Central Hospitals (red dots) in Jyväskylä (Jy CH), Joensuu (Jo CH), Mikkeli (Mi CH), and Savonlinna (Sa CH) serve their own districts (borderlines in black) with 24/7 neuroacutology, CT, and intensive care services. There are also three regional hospitals (green dots) in Iisalmi, Pieksämäki, and Varkaus, serving their own subdistricts. The transfer distances in road kilometers (km) by ambulance to KUH are shown in the flowchart (Fig. 2). Abbreviations: KUH, Kuopio University Hospital; Jy CH, Jyväskylä Central Hospital; Jo CH, Joensuu Central Hospital; Mi CH, Mikkeli Central Hospital; Sa CH, Savonlinna Central Hospital; CT, computed tomography; SAH, subarachnoid hemorrhage; GPS, Global Positioning System; EMS, emergency medical services; HEMS, helicopter emergency medical services; km, kilometer

**Fig. 2:**
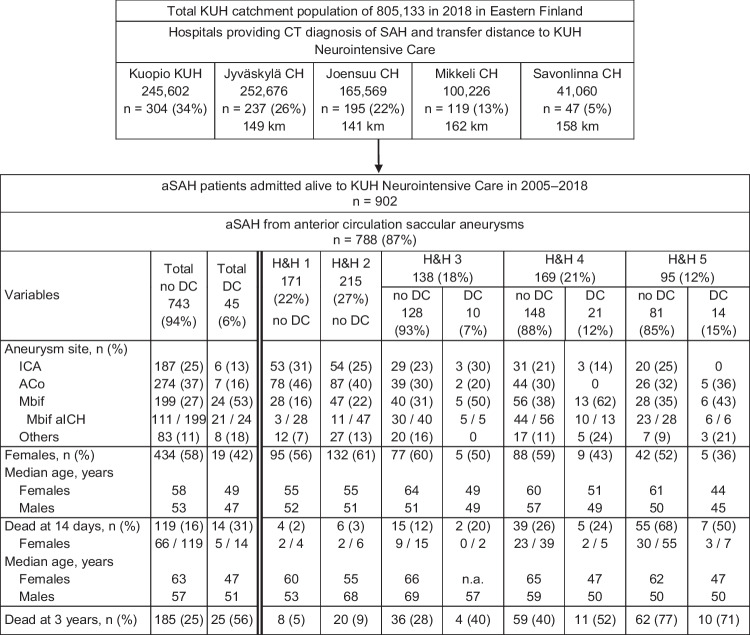
Flowchart. A total of 902 consecutive patients were acutely admitted—within 24 h from the CT diagnosis of the first subarachnoid hemorrhage (SAH)—to the neurosurgical and neurointensive care at the tertiary Kuopio University Hospital (KUH) between 2005 and 2018 from a defined Eastern Finnish catchment population (Fig. 1). The overall KUH catchment area contains four Central Hospitals. The transfer distances in road kilometers (km) by ambulance to KUH are denoted. The 788 (87%) aSAH patients with a ruptured anterior circulation saccular aneurysm, including all 114 posterior communicating artery (PCo) aneurysms, were selected for the present analysis, with regard to the 45 cases of decompressive craniectomy (DC). The 114 (13%) posterior circulation aneurysm cases were excluded, including three DC cases (aneurysm sites: vertebral artery, basilar trunk, P3 of posterior cerebral artery). Abbreviations: CT, computed tomography; SAH, subarachnoid hemorrhage; KUH, Kuopio University Hospital; CH, Central Hospital; km, kilometer; aSAH, aneurysmal SAH; PCo, posterior communicating artery; DC, decompressive craniectomy; H&H, Hunt and Hess scale; ICA, internal carotid artery trunk and bifurcation; ACo, anterior communicating artery; Mbif, middle cerebral artery bifurcation; aICH, intracerebral hemorrhage from ruptured saccular aneurysm; n.a., not applicable

KUH, one of the five university hospitals in Finland, is an academic, non-profit, publicly funded tertiary center, which serves a defined population (805,133 in 2018) in Eastern Finland (Fig. 1). The overall KUH catchment area, during the study period 2005–2018, contained four Central Hospitals with the districts of their own, each with 24/7 neuroacutology, CT services, intensive care, and neurorehabilitation (Figs. 1 and 2). The road transfer distances between KUH and each Central Hospital range from 141 to 162 km.

The Finnish Emergency Response Center handles the calls to the public emergency number 112, including requests for EMS [8, 72]. In case of an EMS call, the dispatcher uses a computer-aided and criteria-based dispatch system to classify the mission to one of four urgency classes and to 46 EMS-specific mission types, based on symptoms, findings, or injury mechanisms.

During the study period, the KUH catchment area (Fig. 1) was served by 74 (1 per 10,900 citizens) advanced level and basic level EMS units and one physician-manned helicopter emergency medical service (HEMS) unit. Dispatching and mission control is Global Positioning System (GPS) based.

### Role of EMS and HEMS in possible acute brain catastrophe in Eastern Finland

The EMS patient care report contained the following time points in minute scale: 112 call, start of mission, arrival in scene, start of transport to hospital, and at hospital. The ambulance personnel assessed the patient’s condition (including Glasgow Coma Scale (GCS)) and preceding course, previous history, and medications. The EMS physician on duty was contacted by phone, and the ambulance personnel were directed to the first hospital with on-line CT. In case of seizure, i.v. benzodiazepine was immediately administered by the protocol. In case of decreased consciousness on arrival to scene or deterioration during transfer, possibly with a dilated pupil, the treatment was guided by the EMS physician. Supraglottic devices could be used to secure the airways. Rapid sequence intubation was performed either EMS physician on scene or in the first hospital.

### KUH NeuroCenter and subarachnoid hemorrhage (SAH) in Eastern Finland

At KUH Neurosurgery, at least two neurosurgeons were on duty at all times, with on-line phone and teleconsultation of digital imaging from the referring hospitals. In principle, all patients with SAH are acutely transferred to KUH for neurointensive care, 4-vessel catheter angiography and/or CT angiography, and neurosurgical and endovascular interventions, virtually regardless of the age or condition on admission, including Hunt & Hess scale (H&H) 4–5 patients [5, 52]. Depending on the patient’s condition and CT findings, intubation (if not performed) and a physician, anesthesiologist, or intensivist attending the patient during the transfer were agreed. Pre-arrival information by HEMS or EMS was given the KUH Emergency area staff.

At KUH, a dedicated team of neurointensivists, neurosurgeons, and interventional neuroradiologists coordinated the aSAH treatment. The KUH Neurovascular Group provided microsurgical or endovascular occlusion of the aneurysm and evacuated significant ICHs with immediate microsurgery. The KUH aSAH protocol in 2005–2018 followed international recommendations, aimed to prevent further brain damage due to re-bleeding, increased ICP (ICP below 20 mmHg; cerebral perfusion pressure at 60–70 mmHg), hydrocephalus, electrolyte disturbances, seizures, cardiac and pulmonary dysfunction, fever, hyperglycemia, and development of delayed brain ischemia. The protocol included extraventricular drainage (EVD), ventricular or parenchymal ICP monitoring, endovascular procedures and intra-arterial nimodipine infusion, and DC. KUH neurointensive care monitoring data is stored in the prospective Finnish Intensive Care Consortium database [78].

### Kuopio intracranial aneurysm patient and family database

The database, prospective since 1995, contains all cases of unruptured and ruptured intracranial aneurysms (IAs) admitted to KUH since 1980. A dedicated, full-time nurse administrates the database, interviews all new IA patients, including their family history, and arranges the follow-ups. The clinical data including prescribed medicines, hospital diagnosis, and causes of death have been derived from national registries, using the Finnish personal codes. We have characterized the aSAH patients, e.g., for 14-day mortality and organ donation [52], three-year outcome [5], shunt-dependent hydrocephalus [1] and shunt revisions [100], depression [39], epilepsy [38], pain [56], psychosis [69], secondary hypertension [47], pre-eclampsia [48], and polycystic kidney disease [66, 67]. The collaboration with UMC Utrecht Neurology and the International Stroke Genetics Consortium is significant.

### Basic study population of the 788 aSAH patients with ruptured anterior circulation sIA

**Fig. 3:**
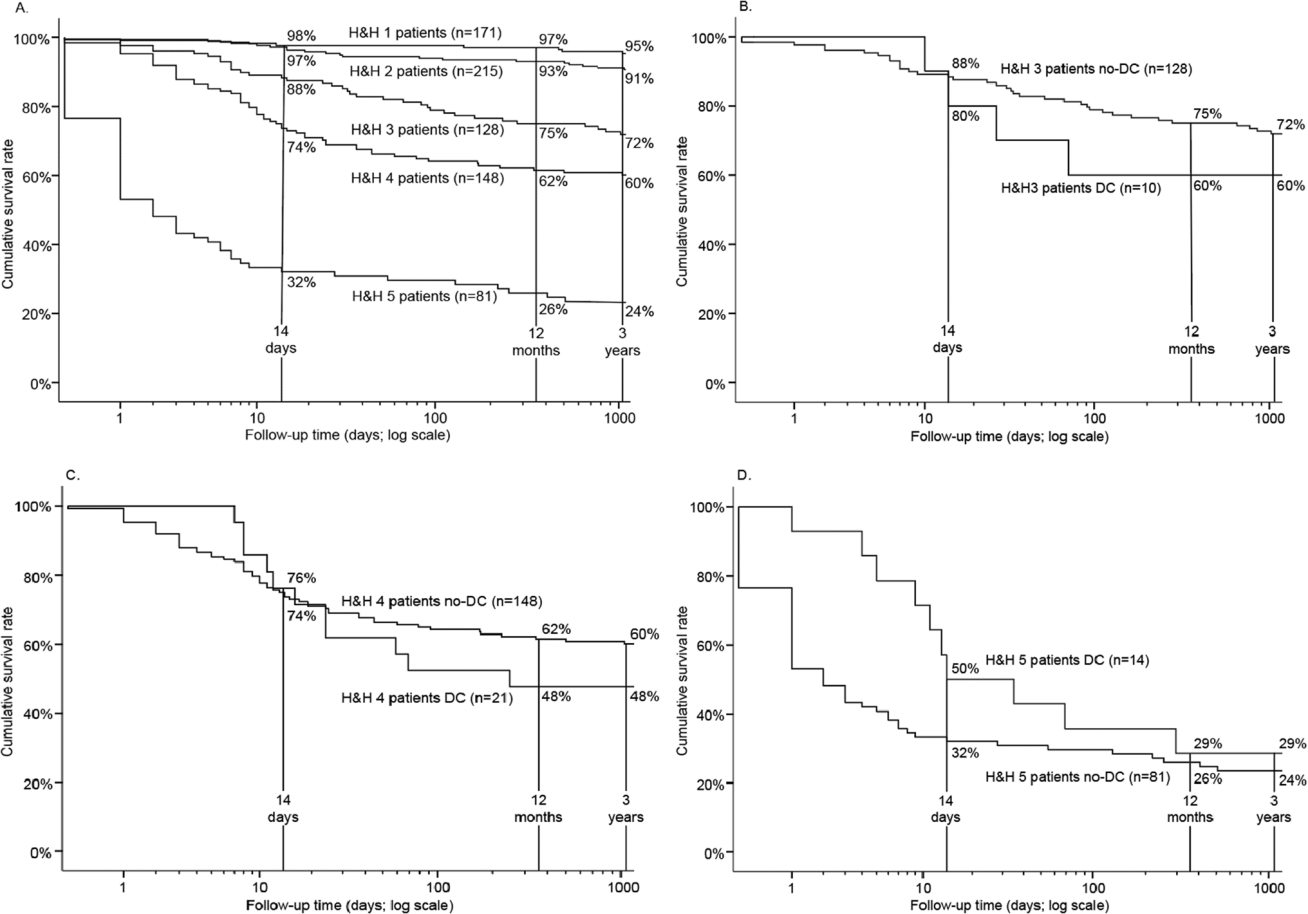
The cumulative survival rates. The cumulative survival rates at 14 days, 12 months, and three years of the 788 patients acutely admitted for the first verified subarachnoid hemorrhage from an anterior circulation saccular aneurysm (aSAH) to the neurosurgical and neurointensive care at the Kuopio University Hospital (KUH) between 2005 and 2018 from its defined Eastern Finnish catchment population. A total of 45 (6%) patients underwent a secondary decompressive craniectomy (DC). The follow-up time is logarithmic to emphasize the early high mortality. **A** H&H 1–5 patients without DC ($n=743$). **B** H&H 3 patients with DC ($n=10$) vs. no DC ($n=128$). **C** H&H 4 patients with DC ($n=21$) vs. no DC ($n=148$). **D** H&H 5 patients with DC ($n=14$) vs. no DC ($n=81$). Abbreviations: aSAH, subarachnoid hemorrhage from anterior circulation saccular aneurysm; KUH, Kuopio University Hospital; DC, decompressive craniectomy

A total of 788 consecutive aSAH patients with a ruptured anterior circulation sIA were acutely admitted, within 24 h from the CT diagnosis of the first SAH, from the defined Eastern Finnish catchment population to the KUH Neurointensive Care Unit from 2005 to 2018 (Figs. 1 and 2). Their clinical lifelines were re-constructed from their clinical data in the Kuopio database and from the national clinical registries until death ($n=210$) or until three years. Their basic variables are in the flowchart (Fig. 2). The cumulative survival rates for the 788 patients at 14 days, 12 months, and 3 years according to the H&H scales (Fig. 2) are in Fig. 3.

### Pilot study population of the 45 DC patients

We chose DC as an indicator of severely escalating intracranial conditions since the aSAH ictus during EMS and neurointensive care. Of the 788 aSAH patients, a total of 45 (6%) underwent a secondary DC, while no primary DCs were performed (Fig. 2, Table 1). The decision of DC was agreed case-by-case between the attending neurointensivists and neurosurgeons, during office hours and duty hours. During the same 14 years (2005–2018), we performed 61 DCs for brain infarction [33] and 56 for TBI [37] in Eastern Finland (population 805,133 in 2018) (Fig. 1).

**Table 1:** Characteristics of the 45 aSAH patients with a secondary decompressive craniectomy (DC) in the neurosurgical and neurointensive care at the tertiary Kuopio University Hospital (KUH) between 2005 and 2018 from its defined Eastern Finnish catchment population (Figs. 1, 2, 3, 4, 5)

Variables	Dead until 3 years $n = 25$ (56%)	$P$ values*	Alive at 3 years $n = 20$ (44%)
Females, $n$ (%) Median age at aSAH, years** Drug-treated hypertension, $n$ (%)	8 (32) 27–44–**49**–58–64 12 (48)	n.s n.s 0.05	11 (55) 30–38–**46**–53–60 4 (20)
Symptoms and condition until the first contact with EMS			
Headache only, $n$ (%) Primary seizure, $n$ (%) Primary unconsciousness, $n$ (%) Median GCS**	3 (12) 6 (24) 21 (84) 3–4–**8**–14–15	n.s 0.02 n.s n.s	6 (30) 0 13 (65) 3–4–**11**–14–15
Condition at KUH arrival			
H&H 3, $n$ (%) H&H 4, $n$ (%) H&H 5, $n$ (%)	4 (16) 11 (44) 10 (40)	n.s n.s n.s	6 (30) 10 (50) 4 (20)
aICH, $n$ (%) Median aICH volume, cm^3**^ aIVH, blood clot, $n$ (%) aIVH, blood sediment only, $n$ (%) Re-bleeds before IA occlusion, $n$ Possibly by symptoms, $n$ (%) Verified by two CTs, $n$ (%)	22 (88) 1–2–**18**–64–135 9 (36) 11 (44) 20 16/20 (80) 4/20 (20)	n.s n.s 0.04 n.s n.a n.s n.s	18 (90) 3–8–**26**–62–105 2 (10) 9 (45) 11 8/11 (73) 3/11 (27)
Anterior circulation sIA site, $n$ (%) ICA ACo Mbif Others Ruptured sIA size, mm**	5 (20) 5 (20) 11 (44) 4 (16) 3–6–**7**–10–20	n.s n.s n.s n.s n.s	1 (5) 2 (10) 13 (65) 4 (20) 3–6–**8**–10–25
Timelines from KUH arrival			
To EVD ($n=42$) and ICP ($n=45$***), hours** To aICH evacuation, hours** To sIA occlusion, hours** To DC, days** To shunt, days ($n$)** From DC to death, days** From DC to cranioplasty, months ($n$)** To death, days**	< 1– < 1–**1**–11–36 < 1– < 1–** < 1**–2–7 < 1–1–**4**–21–64 < 1–1–**2**–3–13 3 and 34 ($n=2$) < 1–4–**8**–43–295 n.a < 1–8–**13**–46–296	n.s 0.01 n.s n.s n.s n.r n.r n.r	< 1– < 1–**3**–29–170 1–2–**4**–11–19 < 1–3–**9**–19–40 < 1–1–**2**–4–8 16–27–**48**–136–150 ($n=6$) n.a < 1–1–**4**–5–8 ($n=18$) n.a
Neurosurgical and endovascular interventions			
EVD, $n$ (%) Microsurgical IA occlusion, $n$ (%) With aICH removal, $n$ (%) Endovascular IA occlusion, $n$ (%) Re-bleed during coiling Re-bleed after coiling Endovascular therapy of brain ischemia, $n$ (%)	25 (100) 11 (44) 6/11 (55) 14 (56) 1/14 (7) 3/14 (21) 2 (8)	0.05 0.01 n.s 0.01 n.s n.s n.s	17 (85) 17 (85) 9/17 (53) 3 (15) 0/3 0/3 3 (15)
ICP 12 h before DC (IQR; 25–50–75%), mmHg DC size, cm^2**^ ICP 12 h after DC (IQR; 25–50–75%), mmHg	11–**14**–16 52–85–**96**–113–175 10–**12**–14	n.s n.s n.s	10–**13**–17 68–84–**104**–114–161 10–**12**–15
Neuro ICU time, days** EVD time, days** Meningitis, $n$ (%) Delayed brain injury, $n$ (%) Mechanical ventilation time, days** Tracheostomy, $n$ (%) Death at Neuro ICU, $n$ (%) Death at KUH ward, $n$ (%) Death at other hospital, $n$ (%)	1–7–**8**–13–19 1–5–**7**–10–15 4 (16) 23 (92) 1–7–**9**–15–35 16 (64) 7 (28) 8 (32) 10 (40)	0.01 n.s n.s < 0.01 n.s n.s n.r n.r n.r	7–10–**12**–17–53 1–4–**7**–9–16 5 (25) 11 (55) 4–6–**9**–12–23 10 (50) n.a n.a n.a
Outcome of 20 survivors after DC			
mRS at 3 years (numbers of scores 0 to 5) Epilepsy only after aSAH, $n$ (%) Depression only after aSAH, $n$ (%) Return to work, $n$ (%)	n.a	n.r	0–5–4–4–5–2 14 (70) 12 (60) 6 (30)

*aSAH* SAH from anterior circulation saccular aneurysm, *DC* decompressive craniectomy, *KUH* Kuopio University Hospital, *EMS* emergency medical services, *GCS* Glasgow Coma Scale, *EVD* extraventricular drainage, *ICP* intracranial pressure, *aICH* aneurysmal intracerebral hemorrhage, *H&H* Hunt & Hess scale, *sIA* saccular intracranial aneurysm, *ICA* internal carotid artery trunk and bifurcation, *ACo* anterior communicating artery, *Mbif* middle cerebral artery bifurcation, *IQR* 25% and 75% range, *aIVH* aneurysmal intraventricular hemorrhage, *IA* intracranial aneurysm, *CT* computed tomography, *ICU* intensive care unit, *mRS* modified Rankin Scale, *n.s.* not significant, *n.r.* not relevant, *n.a.* not applicable

^*^The *P* value column refers to the comparison of the dead (left) and those alive at 3 years (right)

^**^The various variables are expressed by five number, as follows: minimum - 25% quartile -** median** - 75% quartile - maximum. The median values are emphasized by bolding

^***^Three survivors had parenchymal ICP monitoring only

The distribution (IQR; 25–50-75%) of the ICP in each of the 45 patients was analyzed 12 h before and 12 h after the DC (Table 1). A novel, semi-automated image analysis software was used to estimate the area (cm^2^) of the removed skull (Disior Ltd, Helsinki, Finland).

### Individual EMS care and KUH neurointensive care timeline panels for the 45 DC patients

**Fig. 4:**
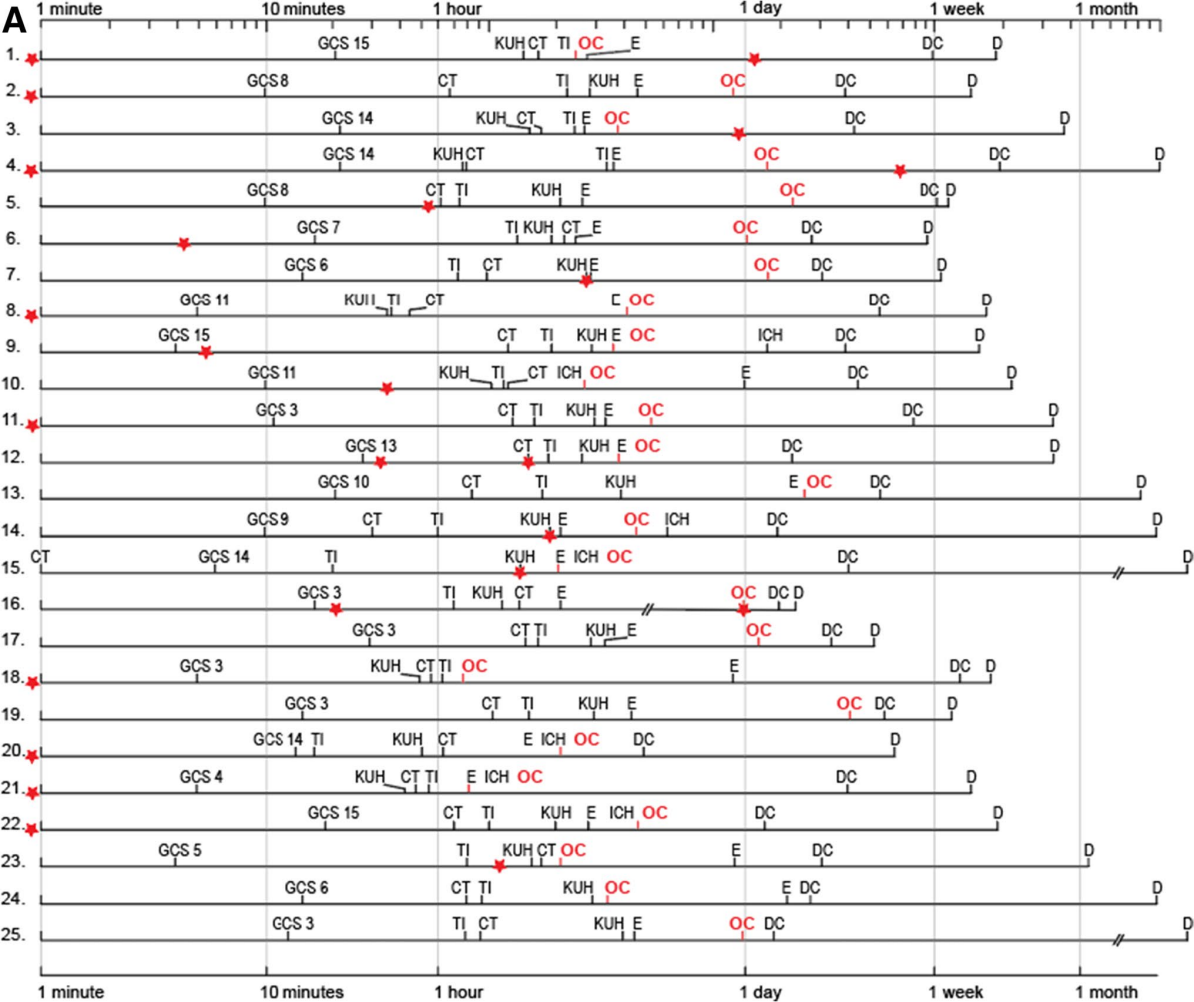

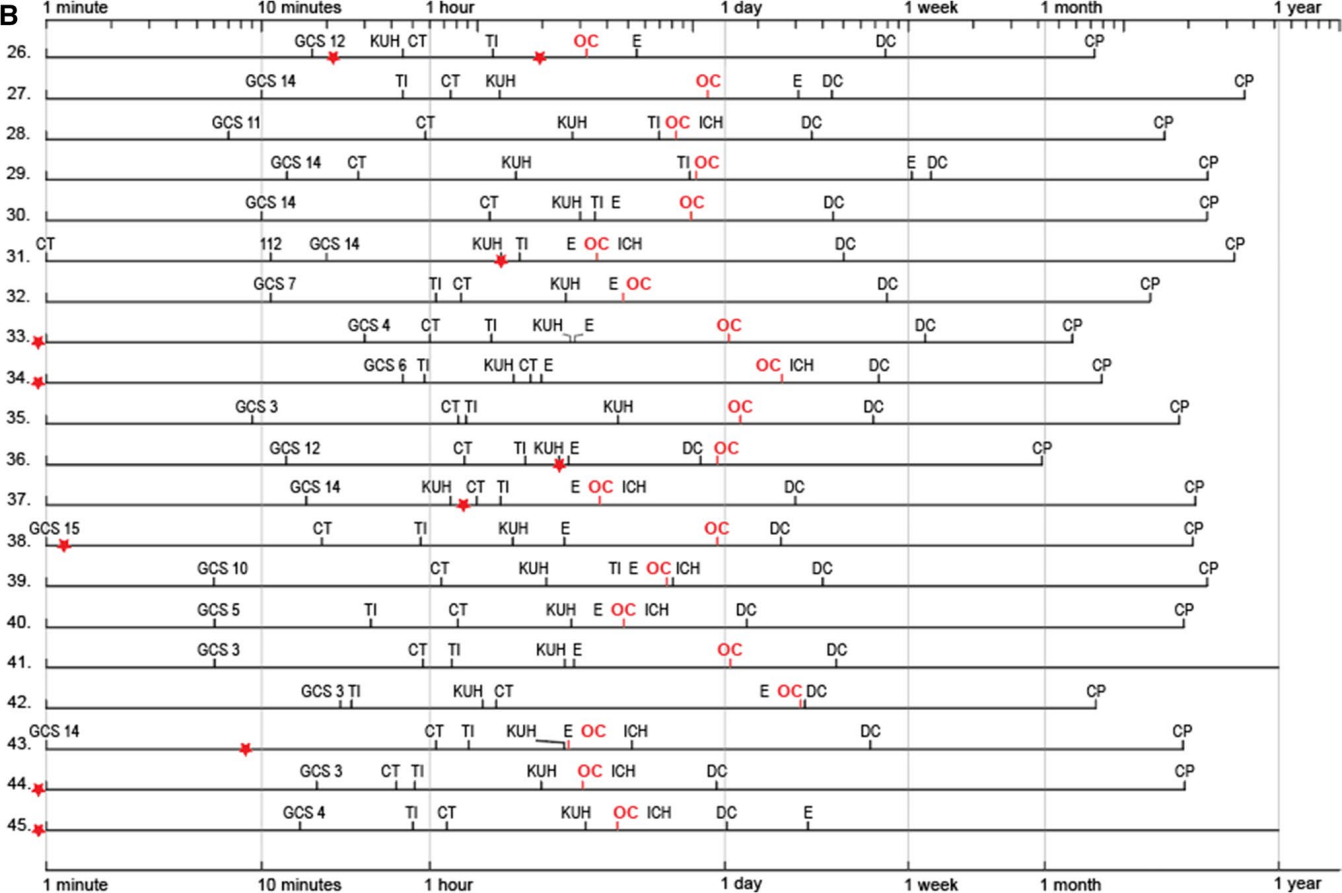
Individual timeline panels of the 45 DC patients. Individual timelines through the emergency care and the neurointensive care of the 45 aSAH patients whose condition required a secondary decompressive craniectomy (DC) during their neurointensive care course at the Kuopio University Hospital (KUH) between 2005 and 2018 from a defined population. **A** The 25 deceased DC patients numbered 1.–25. as in their CT panel in Fig. 5A. **B** The 20 DC survivors numbered 26.–45. as in their CT panels in Fig. 5B. The timelines start from the emergency phone call (112), if not indicated otherwise. The time scale in minutes is logarithmic to emphasize the EMS, transfer, and early KUH phases. The time points of one minute, 10 min, one hour, one day, one week, one month, and one year are indicated by vertical thin lines. The two timeline panels are zoomable to study details. The red star indicates the time points of either suspected (seizure, unconsciousness, dilated pupil suggestive of tentorial herniation) or CT-verified re-bleeds. The time points on the timelines are as follows: 112 = 112 call; GCS = ambulance arrival (Glasgow Coma Scale points 3–15 denoted); CT = diagnostic computed tomography at the first hospital; TI = tracheal intubation; KUH = KUH arrival; E = extraventricular drainage installation and start of ICP monitoring; OC = start of ruptured sIA occlusion; ICH = intracerebral hemorrhage removal; DC = decompressive craniectomy; D = death; CP = cranioplasty. Five patients (15. 27. 31. 38. 43.) came to the first medical assessment on their own. Importantly, all abbreviations above can be identified in the two panels using the find command: for example, the time points of all CTs, EVD installations, sIA occlusions, or decompressive craniectomies. In **B**, however, the find command (CP) shows that two of the 20 survivors (41. 45.) did not receive cranioplasty

**Fig. 5:**
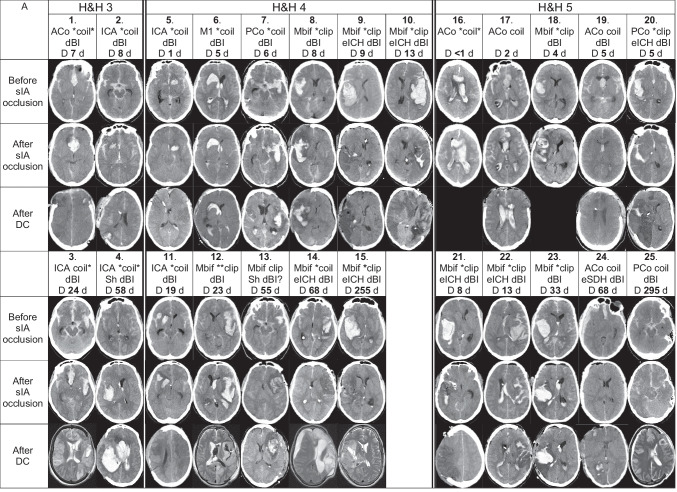

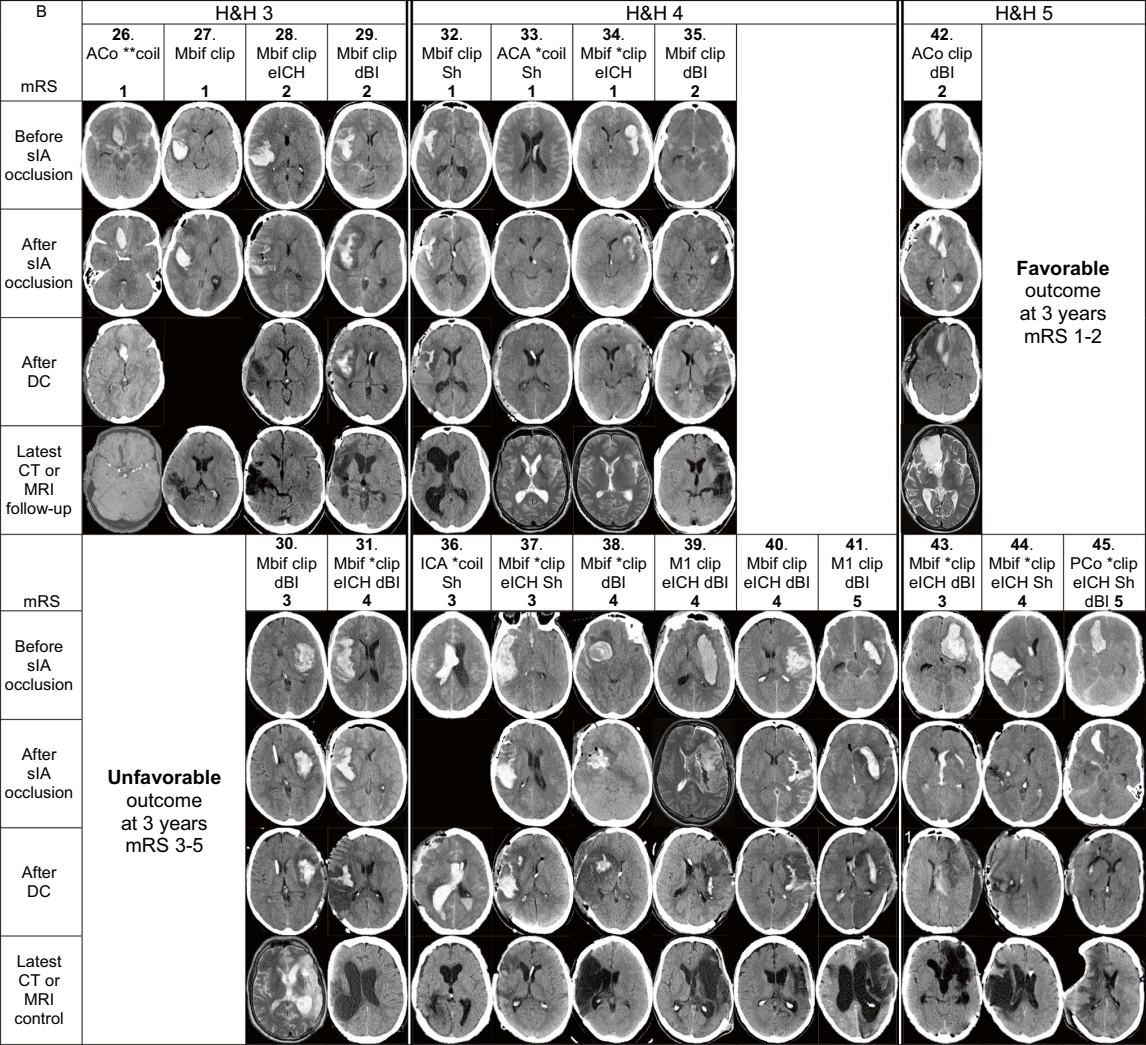
Individual CT/MRI panels of the 45 DC patients. Serial CT scan panels of the 45 aneurysmal subarachnoid hemorrhage (aSAH) patients who underwent decompressive craniectomy (DC) after admission within 24 h from the CT diagnosis of SAH to the tertiary Kuopio University Hospital (KUH). The patients (white data box) and the representative CT scan slices are arranged into vertical columns according to the Hunt & Hess scale (H&H 3–5) on admission. The black areas indicate the lack of CT or MRI scan. The white data box contains the patient number; site of the ruptured saccular intracranial aneurysm (sIA); microsurgical (clip; 28/45) or endovascular (coil; 17/45) occlusion; evacuation of aICH (eICH; 16/45) or aSDH (eSDH; 1/45) during the microsurgical clipping (15/28) or after the endovascular occlusion (2/17); delayed brain injury (dBI) seen here in the third CT or MRI of the patient (34/45). dBI? denotes uncertainty between dBI vs. peri-ICH edema. Asterisk (*) indicates the sIA re-bleeding between the ictus and the sIA occlusion (30/45), either clinically suspected (seizure or worsened condition; 24/45) or verified by two CT scans (6/45). Furthermore, there were four re-bleeds during or after the sIA coiling. **A** Serial CT scan panel of the 25 aSAH patients who died within three years after DC, arranged from left to right according to increasing times (days) from DC to death. For each patient, three CT slices were selected: (1) CT: before clipping or coiling; (2) CT: after clipping or coiling; (3) CT: after DC. Of the deceased DC patients, there were 20 sIA re-bleeds (16 clinical; 4 CT verified) between the ictus and the sIA occlusion. *clip = sIA re-bleed before clipping (9/28); **clip = sIA re-bleed two times before clipping (1/28); *coil = sIA re-bleed before coiling (6/17); coil* = sIA re-bleed during or after coiling (1/17); *coil* = sIA re-bleed before coiling and re-bleed during or after coiling (3/17). Of the 25 patients, two had a ventriculoperitoneal shunt (Sh). **B** Serial CT scan panel of the 20 aSAH patients who survived after DC, arranged from left to right according to the modified Rankin Scale (mRS) within the H&H (3–5) columns. For each patients, four CT slices were selected: (1) CT: before clipping or coiling; (2) CT: after clipping or coiling; (3) CT: after DC; (4) CT or MRI during follow-up at about 12 months. Of the survived DC patients, there were 11 sIA re-bleeds (8 clinical; 3 CT verified) between the ictus and the sIA occlusion. *clip = sIA re-bleed before clipping (7/28); *coil = sIA re-bleed before coiling (2/17); **coil = sIA re-bleed two times before coiling (1/17). Of the 20 patients, six had a ventriculoperitoneal shunt (Sh). Abbreviations: CT, computed tomography; aSAH, subarachnoid hemorrhage from anterior circulation saccular aneurysm; DC, decompressive craniectomy; SAH, subarachnoid hemorrhage; KUH, Kuopio University Hospital; H&H, Hunt & Hess scale; MRI, magnetic resonance imaging; sIA, saccular intracranial aneurysm; clip, microsurgical occlusion; coil, endovascular occlusion; aICH, intracerebral hemorrhage from ruptured anterior circulation saccular aneurysm; eICH, evacuation of aICH; aSDH, acute subdural hemorrhage from aSAH; eSDH, evacuation of aSDH; dBI, delayed brain injury; ICH, intracerebral hemorrhage; Sh, shunt; mRS, modified Rankin Scale; ACo, anterior communicating artery; ICA, internal carotid artery trunk and bifurcation; M1, M1 segment of the middle cerebral artery; PCo, posterior communicating artery; Mbif, middle cerebral artery bifurcation; ACA, anterior cerebral artery

We collected from all available sources (EMS charts; CT, MRI, angiography; intensive care; interventions; hospital case reports) the defined time points (Fig. 4) to reconstruct the clinical timelines for each of the 45 DC patients. The 25 deceased DC patients are numbered 1.–25. (Figs. 4A and 5A) and the 20 surviving DC patients 26.–45. (Figs. 4B and 5B). The timelines allowed to calculate and compare the times passed between any two time points, e.g., from the ambulance arrival to the first CT (minutes), the KUH arrival (minutes), and the sIA occlusion (hours) (Fig. 4, Table 2). The time periods that seemed outliers (very short or very long) were re-checked. Table 3 presents four essential time points according to the weekday, the office hours, and the duty hours.

**Table 2:** Distribution of time periods elapsed among the 45 DC aSAH patients from the ambulance arrival ($n=40$) to the ictus site or from the personal arrival ($n=5$) to the first medical assessment until the occlusion of the ruptured sIA at KUH Neurosurgery. The distributions are expressed as follows in minutes or hours: shortest–25% quartile–**median**–75% quartile–longest. Time exposed to re-bleed before IA occlusion

First hospital $n$ dead–$n$ alive at 3 years	1. From first contact* to first CT at KUH or CH (min)	2. From CT at CH to KUH arrival (h) ($n=29$)***	3. From KUH arrival to start of sIA occlusion (h)	4. From first contact* to start of sIA occlusion (h)
Kuopio KUH** 11 dead–5 alive	28–50–**91**–144–202 **113** vs. **83**	n.r	0.5–2.0–**4.2**–15–41 **2.8** vs. **4.9**	1.2–3.6–**5.5**–17–42 **4.3** vs. **5.9**
Jyväskylä CH 6 dead–5 alive	24–52–**66**–104–125 **66** vs. **73**	1.9–2.5–**2.8**–3.6–6.1 **2.7** vs. **3.1**	0.9–1.2–**4.5**–20–37 **1.6** vs. **18**	4.4–5.5–**7.6**–24–43 **6.0** vs. **21**
Joensuu CH 3 dead–6 alive	7–50–**62**–96–120 **118** vs. **56**	2.0–2.4–**2.9**–3.3–3.7 **2.5** vs. **3.1**	0.1–3.7–**9.2**–22–35 **22** vs. **6.5**	4.4–6.8–**13**–26–38 **27** vs. **10**
Mikkeli CH 4 dead–4 alive	15–30–**67**–83–117 **69** vs. **66**	2.0–2.3–**3.1**–4.0–5.2 **3.3** vs. **2.7**	2.1–3.7–**15**–19–25 **16** vs. **9.1**	7.2–7.6–**19**–24–29 **22** vs. **12**
Savonlinna CH 1 dead	**91**	**3.2**	**64**	**69**
45 DC patients 25 dead–20 alive	**66** **80** vs. **64**	**2.9** **2.8** vs. **3.1**	**4.9** **4.5** vs. **9.2**	**7.9** **7.6** vs. **13**
Tracheal intubation		CH patients ($n=29$)***		n.r
GCS 3–8 ($n=22$)				
13 dead–9 alive	5/13–4/9	5/8–4/7	3/13–1/9	
GCS 9–12 ($n=8$)				
4 dead–4 alive	2/4–0/4	2/2–1/3	0/4–3/4	
GCS 13–15 ($n=15$)				
8 dead–7 alive	1/8–1/7	4/4–3/5	3/8–3/7	

The hours are expressed with two digits. The times are expressed with five numbers, as follows: shortest - 25% quartile - **median** - 75% quartile - longest. The median values are emphasized by bolding in the five-number sequences, as well as, in the median time comparison between the dead and alive

*DC* decompressive craniectomy, *aSAH* subarachnoid hemorrhage from anterior circulation saccular aneurysm, *sIA* saccular intracranial aneurysm, *KUH* Kuopio University Hospital, *IA* intracranial aneurysm, *CT* computed tomography, *CH* Central Hospital, *GCS* Glasgow Coma Scale, *HEMS* helicopter emergency medical services, *n.r.* not relevant

^*^The first contact: see Table 1 legend

^**^The 16 KUH patients include 6 patients who came from the CH districts directly by HEMS to the first CT at KUH

^***^29 CH patients arrived to KUH

**Table 3:** The time points of the timelines of 45 decompressive craniectomy (DC) aSAH patients from the ictus to the sIA occlusion at KUH Neurosurgery, according to the weekday and the office hours vs. duty hours. The weekdays are defined according to the hours presented in the table, with 40 office hours and 128 duty hours in the week. The KUH arrivals clustered on Mondays ($n=12$), because the first EMS contacts also clustered on Mondays ($n=12$)

		Monday 8–8 Tue		Tuesday 8–8 Wed		Wednesday 8–8 Thu		Thursday 8–8 Fri		Friday 8–24 Fri		Saturday 0–24 Sat	Sunday 0–8 Mon
Time points	$N$(%)	Office 8–16	Duty 16–8	Office 8–16	Duty 16–8	Office 8–16	Duty 16–8	Office 8–16	Duty 16–8	Office 8–16	Duty 16–24	Duty 0–24 Sat	Duty 0–8 Mon
EMS contact	45	10*	2	4	2	5	5	2	1	2	1	6	5
First CT	45	8*	4	4	2	4	6	1	2	1	2	6	5
KUH arrival aICH	45 40/45 (89)	8* 7	4 4	3 3	3 1	3 3	6 5	2 2	2 2	1 1	2 2	5 5	6 5
Clipped sIA aICH Evacuated	28 (62) 27/28 (96) 15/27 (56)	3 3 2	2 2 1	3 3 2	0 0 0	2 2 0	3 3 3	3 3 2	1 0 0	2 2 0	1 1 1	4 4 1	4 4 3
Coiled sIA aICH Evacuated	17 (38) 13/17 (76) 1/13 (8)	1 1 0	4 4 0	2 0 0	1 1 0	1 0 0	2 1 0	3 3 1	0 0 0	0 0 0	0 0 0	1 1 0	2 2 0

*DC decompressive craniectomy, *aSAH* subarachnoid hemorrhage from anterior circulation saccular aneurysm, *sIA* saccular intracranial aneurysm, *aICH* intracerebral hemorrhage from ruptured sIA, *KUH* Kuopio University Hospital, *EMS* emergency medical services, *CT* computed tomography, *Clipped* microsurgical occlusion, *Coiled* endovascular occlusion

*The bolded numbers emphasize the clustering of the patients on Monday

### Possible re-bleeds until the sIA occlusion among the 45 DC patients

The patients’ timelines were analyzed for possible re-bleeds until the sIA occlusion (Figs. 4 and 5, Tables 1 and 4). The verified re-bleeds were seen in two consecutive CT scans, and the suspected re-bleeds were considered as worsening of the clinical condition (seizure, unconsciousness, dilated pupil). The CT-verified re-bleeds during or after the sIA occlusion were also registered.

**Table 4:** The patients with one or two suspected ($n = 24$) and/or verified ($n = 11$) re-bleeds among the 45 DC aSAH patients from the ictus to the period after sIA occlusion at KUH Neurosurgery. The suspicion of re-bleed is derived from the documented worsening of the clinical condition (seizure, unconsciousness, dilated pupil suggestive of tentorial herniation) after the ictus. The verified re-bleed means increased hemorrhage between two consecutive CT scans

	24 patients with one or two suspected re-bleeds		11 patients with one or two verified re-bleeds			
45 DC patients	1. After ictus to first contact	2. First contact to first CT	3. First CT to KUH CT	4. KUH CT to sIA occlusion	5. During sIA occlusion	6. After sIA occlusion
25 dead with 24 re-bleeds	$n = 9$ 2. 8. 11. 18. 20. 21. 22. + 1. + 4.	$n = 7$ 5. 6. 9. 10. 23. + + 12. + 16.	$n = 4$ 7. 14. 15. + + 12.	None	$n = 1$ + 16 +. coiling	$n = 3$ 3. coiled + 1 +. coiled + 4 +. coiled
20 alive with 11 re-bleeds	$n = 4$ 33. 34. 44. 45.	$n = 4$ 37. 38. 43. + + 26.	$n = 2$ 31. 36.	$n = 1$ + + 26.	None	None
All	$n = 13$	$n = 11$	$n = 6$	$n = 1$	$n = 1$	$n = 3$

The CT panels in Fig. 5 indicate the re-bleeds among the 25 deceased DC patients (numbers 1.–25.) and the 20 alive DC patients (numbers 26.–45.). The timelines in Fig. 4 indicate the approximate time points of the re-bleeds. Two re-bleeds occurred in 5 patients, indicated in the table with two + marks (+ 1 + . + 4 + . +  + 12. + 16 + . +  + 26.)

*DC* decompressive craniectomy, *aSAH* subarachnoid hemorrhage from anterior circulation saccular aneurysm, *sIA* saccular intracranial aneurysm, *KUH* Kuopio University Hospital, *CT* computed tomography, *coil* microsurgical occlusion

### Individual serial CT/MRI scan panels for the 45 DC patients

The KUH digital image archive (PACS) is linked to the four Central Hospitals in Eastern Finland (Figs. 1 and 2). We were able to review and retrieve all CT scans, MRI scans, and angiographies in the five digital archives of the 45 DC patients, using their personal identity codes. For each DC patient, we chose three representative slices (before and after the sIA occlusion and after DC), and for the 20 survivors, the fourth slice at about 12 months (Fig. 5). We recorded the date and the clock time of the brain imaging study in which our experienced neuroradiologists saw the first signs suggestive of brain ischemia.

### Literature search for published individual timeline panels or serial CT/MRI panels for all patients in acute brain catastrophe cohorts

We searched PubMed for English articles from 2000 to 2022 on human brain insult cohorts (brain infarction, ICH, IVH, SAH, and TBI) with (i) *all patients’ individual timeline illustration panels* since the ictus through EMS care, neurointensive care, and rehabilitation, to the outcome at one to three years (Table 5). As of 6/2022, the 218 hits contained only one (our own) article with a timeline panel [100]. Instead, four known articles with the timeline panels [48, 50, 51, 99] were not found because “timeline” and “lifeline” were missing in the abstracts. We also searched for articles on (ii) *all patients’ individual serial CT/MRI image panels* since the ictus until one to three years, disclosing final brain injury areas and atrophy (Table 6). Our search words are presented in Tables 5 and 6. Thesaurus (thesaurus.com) was reviewed for synonyms for the search words.

**Table 5:** Literature search for English articles from 2000 to 2022 on brain insult cohorts with *all patients’ individual timeline or lifeline panels*

Search words	Hits	Articles with timeline or lifeline panels
((timeline OR lifeline OR workflow) AND patient)	17,470	
AND (brain infarct)	48	0
AND ((intracerebral hemorrhage) OR ICH)	69	0
AND ((intraventricular hemorrhage) OR IVH)	3	1 [100]
AND ((subarachnoid hemorrhage) AND aneurysm*)	10	1 [100]
AND ((traumatic brain injury) OR TBI OR (brain trauma))	84	0
AND (decompressive craniectomy)	4	0
Total	218	1

**Table 6:** Literature search for English articles from 2000 to 2022 on brain insult cohorts with *all patients’ individual serial CT/MRI panels*, not just representative examples

Search words	Hits	Articles with serial CT/MRI panels
((CT OR MRI) AND (serial OR chronologic OR consecutive OR consequent OR sequential OR subsequent OR successive) AND (panel OR collection OR composition)) AND patient	5947	
AND (brain infarct)	155	0
AND ((intracerebral hemorrhage) OR ICH)	204	0
AND ((intraventricular hemorrhage) OR IVH)	36	0
AND ((subarachnoid hemorrhage) AND aneurysm*)	54	0
AND ((traumatic brain injury) OR TBI OR (brain trauma))	122	0
AND (decompressive craniectomy)	12	1 [106]
Total	583	1

### Statistical methods

The categorical variables were expressed in proportions, and the *χ*^2^ test was used for comparisons. The continuous variables were expressed in medians, quartiles, and ranges, and the Mann–Whitney $U$ test was used for comparisons. The time periods between two defined time points were expressed in minutes, hours, days, or months. Their distributions were presented with five times, as follows: shortest–25% quartile–**median**–75% quartile–longest (Tables 1 and 2). The Kaplan–Meier analysis was used to calculate the cumulative mortality rates. In the 45 DC patients (25 dead and 20 survivors), univariate analysis was used to identify factors that associated with the death. Factors associated with a favorable outcome (modified Rankin Scale (mRS) 0–2) and return to work were also searched. *P* values < 0.05 were considered significant. We used the SPSS 27 statistical software (SPSS, Inc., Chicago, IL).

### Ethical aspects

The KUH Research Ethics Committee approved the study. The KUH Neurosurgery IA Study Group had received a written informed consent from all patients in the database. The Ministry of Social Affairs and Health of Finland approved the data fusion from the national registries. The patients of the study cohort were not contacted during the study. In this article, we present *pseudonymized* data only on the 45 DC patients. We excluded from the timelines, CT/MRI slice panels, tables, and texts the following data: name; gender; date, month, and year of aSAH; clock times; time period lengths (except the time to death in Fig. 5A). The Kuopio IA Database does not contain face photos. The CT and MRI slices, three or four per patient (Fig. 5), do not allow individual face recognition. Overall, the data presented does not yield *the correct attribution to an individual patient* (name, personal identity code, face photo or video) without the additional information strictly kept by us within the KUH Information System.

## Results

### 45 DC patients vs. 743 no-DC patients

The 45 DC patients were younger (median age 47 years; $P <0.05$) and more often males (58%; $P$ 0.03) than the 743 (94%) no-DC patients (Fig. 2). Of the 45 DC patients, 25 (56%) died (patients 1.–25.) and 20 (44%) were alive (patients 26.–45.) at three years (Fig. 3). Figure 5 presents the serial CT or MRI slices for each of them, according to the H&H grades (3–5) on admission, as well as the mRS at 3 years for the 20 survivors. The MCA bifurcation sIA ruptured in 24 (53%) DC patients, in all but one causing an aICH ($n=23/24$; 96%) (Figs. 2 and 5, Table 1). In the 357 (45%) H&H 3 to 5 patients with no DC (Fig. 2), 124 MCA bifurcation sIAs (35%) had ruptured, almost equally with aICH ($n=97/124$; 78%).

### From the ictus to the KUH arrival

The site of SAH ictus, with vs. without eyewitnesses, was home ($n=19$; 42% vs. 7; 16%), work ($n=4$; 9% vs. 1; 2%), or other ($n=11$; 24% vs. 3; 7%). The first recorded symptoms and/or signs were headache alone ($n=9$; 20%), seizure ($n=6$; 13%) or collapse ($n=34$; 76%). Five (11%) patients (15. 27. 31. 38. 43.) came to the first hospital and CT on their own; only one of them (patient 15.) died (Figs. 4 and 5). For 40 (89%) patients, the ambulance came to the site after the 112 call but in only 3 (8%) instances called by the patient (6. 26. 34.). The time in minutes from the 112 call to the ambulance arrival was distributed rather equally in the five hospital districts (Figs. 1 and 2, Table 2). At the first contact, GCS was distributed as follows: 13–15 ($n=15$; 33%), 9–12 ($n=8$; 18%), and 3–8 ($n=22$; 49%) (Fig. 4). The first contact clustered on Monday (Table 3), without obvious reasons.

Table 2 presents the distributions of the successive time periods according to the hospital districts. The time periods were checked for possible time point errors and for extended lengths (outliers) (Fig. 4, Table 2). Among the 22 (49%) patients with GCS 3–8 on ambulance arrival, the times to tracheal intubation (3–46–**66**–94–217 min) seemed prolonged in five (23%) patients: 138 min, 138 min, and 152 min with GCS 3; 119 min with GCS 7; 217 min with GCS 8; all five patients died (Fig. 4).

### Suspected or verified re-bleeds until the sIA occlusion

Figure 4 illustrates the approximate time points (red asterisk) for the re-bleeds on the individual timelines, and Table 4 presents them in the six sequential time periods. Importantly, 13 (29%) patients seemed to have *a re-bleed before the 112 call* (Fig. 4, Table 4). Before the sIA occlusion, a total of 29 (64%) DC patients had re-bleeds, 19 (76%) of the 25 deceased patients and 10 (50%) of the 20 surviving patients (Figs. 4 and 5, Tables 1 and 4).

### EVD and ICP monitoring

A total of 42 (93%) DC patients received an EVD with intraventricular ICP monitoring, in a median of five hours since the ambulance arrival to the ictus site (Fig. 4). An EVD was placed in 19 (45%) patients before the sIA occlusion and in 13 (31%) patients during the sIA clipping. Antibiotic prophylaxis (cefuroxime or cloxacillin) i.v. was routine. EVD revisions were performed in 14 (56%; $P$ 0.01) of the 25 deceased but in only three (18%) of the 17 survivors. The median EVD duration was seven days in both groups (Table 1). Clinical meningitis was diagnosed in 9 (21%) of the 42 patients, in association with the EVD duration (median 11 vs. 8 days; $P < 0.01$).

### Timing of the sIA occlusion in the 45 DC patients

CT angiography (CTA) at KUH was the primary method (43/45; 96%) for the diagnosis of ruptured sIA. Table 3 presents the 45 sIA occlusions (28 (62%) microsurgical vs. 17 (38%) endovascular) and the 16 (36%) aICH evacuations according to the working days and weekend days, office hours, and duty hours. Overall, 20 (44%) occlusions took place during office hours and 25 (56%; 10 endovascular) in the duty hours, including 11 (24%; 3 endovascular) during weekend days.

### Occlusion of 45 sIAs and evacuation of 16 aICHs

The 28 (62%) clipped sIAs were somewhat larger (n.s.) (3–7–**9**–13–25 mm; nine (32%) over 10 mm) than the 17 (38%) coiled ones (3–6–**7**–9–10 mm) (Table 1). Of the 28 clipped sIAs, 23 (82%) were MCA bifurcation sIAs, all but one with aICH (Fig. 5, Table 1). Of the 40 aICHs, 15/40 (38%; see 14. below) were exposed during the sIA clipping, but in only 11/15 (73%) a near-total aICH evacuation (patients 9. 10. 15. 21. 22. 28. 34. 39. 40. 43. 44.) was achieved (Fig. 5). No primary DCs were performed. The methods to check the sIA occlusion and open branches were micro-Doppler and indocyanine green (ICG) angiography. Postoperative angiography was obtained in 19 (68%) of the 28 clipped sIAs. One MCA bifurcation sIA with aICH (patient 14.) was first coiled and then evacuated (Fig. 5).

Among the 17 (38%) endovascularly occluded sIAs, six (35%) were on the anterior communicating artery (ACo) and six (35%) on the internal carotid artery (ICA) (Fig. 5). Of the 17 patients, four (24%) had a CT-verified re-bleed (Fig. 5): one (patient 16.) due to catheter perforation and three (patients 1. 3. 4.) with slight residual filling.

### Delayed brain injuries in the 45 DC patients

A total of 34 (76%) patients developed delayed brain injuries of various sizes, regarded as ischemic (Fig. 5), 11/20 (55%) survivors and 23/25 (92%) deceased. Of the 34 delayed brain injury patients, 18 (53%) had a ruptured MCA bifurcation sIA, all but one with aICH (Fig. 5). At least 10 (29%) delayed brain injuries were large or even hemispheric, in 2 survivors (41. 45.) and in 8 deceased (3 10 11 12 15 20 21 25). In 7 (21%; patients 9. 18. 23. 31. 38. 39. 40.) delayed brain injury patients, all with aICH and clipped MCA bifurcation sIA, an M2 branch was found occluded in the postoperative CTA or digital subtraction angiography (DSA). Only two (29%) of the seven M2 occlusions could be anticipated by micro-Doppler or ICG angiography. Intra-arterial nimodipine infusion was administered in 5 patients (11%; patients 3. 11. 27. 32. 39.). In the neurointensive care, it was at first difficult to distinguish between perihematomal edema, brain edema, and delayed ischemic brain injury. The median time from the EMS call to the apparent ischemic injury in CT or CT perfusion was 68 h. The time from the EMS call to the ambulance arrival did not associate with the development of brain ischemia (Fig. 4).

### ICP and secondary DC

Overall, 14 (31%) of the 45 DC patients had a dilated pupil at some phase of the timelines (Fig. 4). The median time from the KUH admission to the DC was 65 h (Fig. 4, Table 1). Three (7%) patients (1. 5. 14.) were treated with hypothermia but none with barbiturate-induced coma. Of the 45 DCs, 42 (93%) were unilateral and 3 (7%) were frontal. The DC areas in the 25 deceased (52–85–**96**–113–175 cm^2^) and in the 20 survivors (68–84–**104**–114–161 cm^2^) were similar (Table 1); the 11 smallest sizes (IQR 25%; 52 to 84 cm^2^) were considered as small. ICP values (1 per 2 min) distributed similarly in the 12-h recordings before and after the DCs (Table 1). There were five (11%) DC complications: four (9%; patients 2. 24. 25. 35.) tiny subcortical ICHs in the DC area (Fig. 5) and one (patient 4.) subgaleal hematoma.

### Factors associated with death among the 45 DC patients

Of the 25 (56%) deceased patients, 18 (72%) patients died in the ward of KUH or another hospital after the termination of neurointensive care due to poor prognosis. The following factors associated with death as compared to the 20 survivors (Table 1): hypertension (48% vs. 20%); seizure at ictus (24% vs. 0%); aIVH blood clot (36% vs. 10%); time from KUH arrival to aICH evacuation (1 vs. 4 median hours); endovascular sIA occlusion (56% vs. 15%); Neurointensive Care Unit (NICU) time (8 vs. 12 median days); delayed brain injury on CT or MRI (92% vs. 55%). The cohort was too small for multivariate analysis.

### Outcome of the 25 surviving DC patients

Among the 20 (44%) DC survivors, six (30%; patients 32. 33. 36. 37. 44. 45.) received a shunt in a median of 49 days, all after DC (Fig. 5B). Of the 20 DC survivors, 18 received a cranioplasty (15 own frozen bone flaps; two titanium meshes; one bioactive glass). There were 15 (75%) CT and five (25%) MRI scans at about one year (Fig. 5B). They reflected (i) the lost brain areas caused by aICH and (ii) injured brain areas caused by ischemia (final brain tissue outcome).

### Favorable outcome (mRS 1 or 2) in the nine surviving DC patients at three years

At three years, nine (45%; median 47 years) of the 20 DC survivors had a favorable clinical outcome (mRS 1 or 2) (Fig. 5B, Table 1). Antiepileptic medication, started in six of the nine (67%) survivors, was later discontinued in three (50%) for being seizure-free. Six (30%; patients 26. 27. 28. 32. 33. 34.) survivors returned to work (median age 49 years), all with a cranioplasty and two (33%) with a shunt. Their characteristics were GCS at first contact 4, 6, 7, 11, 12, 14 (four (67%) intubated before KUH); 3/6 (50%) re-bleeds before sIA occlusion; 3 (50%) with H&H 3 and 3 (50%) with H&H 4; no aIVH clot; 5/6 (83%) with aICH (median 25 cm^3^) of which 2/5 (40%; patients 28, 34) evacuated; median ventilation time 11 days; 1/6 (17%) with a tracheostomy. None of the 6 returnees had areas of new brain infarction adjacent to the bleeding site nor elsewhere (Fig. 5B).

### Unfavorable outcome (mRS 3 to 5) in the 11 surviving DC patients at three years

At three years, 11 (55%; median 45 years) survivors had an unfavorable outcome, two (18%; patients 41. 45.) of whom were in hospice care, nine (82%) with a cranioplasty and four (36%) with a shunt (Fig. 5B, Table 1). Re-bleed occurred in seven of 11 (64%) before the sIA occlusion. All 11 had an aICH (median 43 cm^3^), and seven (64%) of them evacuated. Their median time to DC was two days, as compared to five days in those with favorable outcome. Their median ventilation time was nine days, and eight of 11 (73%) had a tracheostomy. Ischemic brain lesions developed in eight (73%) patients adjacent to the primary aICH (Fig. 5B).

## Discussion

### Individual timeline and serial CT/MRI panels of the 45 aSAH patients with DC during neurointensive care

We chose secondary DC (DC here) as an indicator of escalating intracranial conditions since the aSAH ictus during EMS and neurointensive care of the human CNS system, suffering from and reacting to aSAH that forces arterial blood into subarachnoid spaces (aSAH) [4, 9, 41, 42] and possibly into ventricles (aIVH) [7, 14] and brain tissue (aICH) [15]. We compiled (i) individual timeline panels since the EMS call (Fig. 4) and (ii) serial CT/MRI slice panels (Fig. 5), for the 25 deceased (1.–25.) and for the 20 survivors (26.–45.).

Our study illustrated for the clinician readers the following:

- the swiftness (“Time is Brain”) since the 112 call and possible outliers during the EMS care followed by KUH neurointensive care until the sIA occlusion,
- the sites and sizes of aICHs and aIVHs,
- the development of brain edema, perihematomal edema, and ischemic brain injuries, and.
- the extent of brain injuries and brain atrophy at about 12 months after aSAH (*brain tissue outcome)* in the 20 survivors (“what brain tissue is left to live and try back to work”)—not just mRS 0 to 5 [65, 84] (*patient outcome*).

In our eyes, the possibility to track individual patients or patient groups in the panels essentially enhances the evaluation of their clinical courses, in support of Personalized Medicine. We find that our two timeline panels and two serial CT/MRI panels present quickly much more individual information than any reasonable amount of words, figures, and graphs would convey.

### “Time is Brain”—brain tissue spared or lost in brain infarction vs. aSAH

Minutes count from the first signs of brain ischemia to EMS call to ambulance to hospital to CT to thrombolytic or endoarterial recanalization—to prevent or minimize permanent brain tissue injury with swift and honed logistics [29], also with mobile stroke units [107]. *The key time points and periods* (onset-to-hospital, onset-to-imaging, door-to-needle, door-to-artery, and onset-to-treatment) to the recanalization are defined and recorded (see Table 2) [29, 92]. *The loss of brain tissue per time unit* is derived from the final brain infarct volume on CT/MRI divided by the onset-to-recanalization time [87]. Oddly, some patients have poor outcomes despite small brain infarcts after endovascular recanalization while some with larger infarcts do well [27].

In aSAH, our novel timeline panels (Fig. 4) indicate several time periods that *by extended durations* might (i) reduce the final brain volume and (ii) increase the brain age [21]: 112 to ambulance arrival (seizures, aspiration, hypoxia), 112 to CT (uncertain diagnosis), 112 to intubation and ventilation (aspiration, hypoxia), 112 to EVD (increased ICP, ischemia, brain edema), 112 to neurointensive care (suboptimal monitoring and therapy), 112 to sIA occlusion (exposure to re-bleed, aICH and aIVH), 112 to aICH removal (increased ICP, neuroinflammation), and 112 to DC (increased ICP, ischemia, brain edema).

### Construction and optimization of the individual timeline panels

We have previously published timeline panels from the Kuopio IA Database. Importantly, each panel appeared in a journal page for full attention, not in the Supplements. In *Tervonen *et al*.* (2021), the panel of 101 shunted aSAH patients showed on *a logarithmic day axis* (i) when their adjustable valve shunts were installed and (ii) when and why 25 (25%) were later revised [100]. In *Kurtelius *et al*.* (2019b), the timelines on *a linear age axis since birth* indicated at which age aortic aneurysms were detected, 48 (1.1%) in the 4253 sIA patients and 17 (14%) in the 125 fusiform IA patients [51]. In *Kurtelius *et al*.* (2019a), the risk of sIA was analyzed in the 48 children of the 18 couples with both parents as verified sIA carriers. Six sIA family trees were illustrated with the timelines of two or three generations since the birth on *a linear calendar year axis* (1920–2020) [50]. In *Kotikoski *et al*.* (2021), *the linear age timelines* of 22 female sIA patients indicated when pre-eclampsia was diagnosed in relation to the diagnosis of unruptured sIA, aSAH, and hypertension [48].

Here, the 45 timelines started from the 112 call and ended at death (Fig. 4A) or cranioplasty (Fig. 4B). Their manual creation was arduous, picking up time points, events, and interventions from various sources, partly digital since 2004 (KUH patient files, PACS, and neurointensive care monitoring data), but also from copies of original reports and charts.

The 45 timelines were trimmed to be (in our eyes) readable (abbreviations on the time points), distinguishable (line spacing), scalable (time axis), and zoomable while fitting one journal page. The time axis here is logarithmic (Figs. 3 and 4) so that (i) the EMS events, KUH arrival, EVD, sIA occlusion, and aICH removal (minute scale) could be distinguished from (ii) the neurointensive care period with DC (day scale) and (iii) the follow-up time until death (day scale) or three years (month scale). The time points of *suspected or verified re-bleeds* (red) and *sIA occlusions* (red) were highlighted to illustrate the exposure times. The sequential time intervals (Table 2) were not labelled with colors because that (in our hands) rendered the timelines unreadable.

One challenge was to decide where to place the start line of the timelines. Here, they start from the left 112 call line (zero-minute line) and proceed to the right at different lengths for the individual time periods. The zero-minute line could be placed at another time point, such as intubation, dilated pupil, suspected re-bleed, arrival at KUH, sIA occlusion, or DC. Then, *the divided timeline* proceeds *backwards* until the 112 call and *forwards* until death or three years, underscoring, e.g., the times of exposure to re-bleeds. Finally, our timeline panels would benefit from *simple animation tools—*including a vertical line movable along the time axis to compare the time points.

### Construction and optimization of the individual serial CT/MRI slice panels

Acta Neurochirurgica has published our CT slice panels, each in a journal page for full attention [5, 100]. In *Tervonen *et al*.* (2021), in the 101 shunted aSAH patients, their 101 CT slices before the shunt illustrated blood clots and sediments remaining in the lateral ventricles. The 101 CT slices were arranged according to (i) EVD ($n=82$) or no EVD ($n=19$), (ii) the days from aSAH to the shunt, and (iii) revision ($n=25$) or no revision ($n=76$). In *Autio *et al*.* (2021), the primary CT slices of 120 survivors of poor grade aSAH survivors were arranged according to their mRS (0 to 5) at three years. A total of 71 (59%) patients had aICH, the volume of which significantly predicted mRS at three years.

The KUH PACS is linked to the four Central Hospitals in Eastern Finland (Fig. 1). We were able to review and retrieve all CT and MRI scans in the five digital archives of the 45 DC patients, using their personal identity codes. For each DC patient, we chose three representative slices (before and after sIA occlusion; after DC), and for the 20 survivors the fourth slice at about 12 months (Fig. 5). There are altogether *151 slices* in the two panels. Many adjustments were necessary to create (in our eyes) two readable and evaluable panels, each fitting a journal page. All 45 DC patients were placed into three *vertical columns* (H&H 3, 4, or 5 on admission). The 20 survivors were also placed into *horizontal rows* according to their mRS scale (0 to 5) at three years (Fig. 5B). Each CT slice per time point was selected to show the largest aICH or aIVH volume, perihematomal edema, and thereafter the largest area of brain infarction. The CT slice after DC also reflected the largest DC diameter. There was shortage of MRI scans, none obtained close to the admission. The addition of a 3D CTA image of each ruptured sIA would crowd the panels. CT perfusion images [89] were unreadable in small size.

### Pseudonymization of the individual timelines and serial head CT/MRI slice panels

The categorical concern about the patients’ privacy is obviously one reason not to present their individual timeline and serial brain image panels in the articles on acute brain insults. In this article, we present *pseudonymized* data only on the 45 DC patients. The data presented here does not enable *the correct attribution to an individual patient* (name, personal identity code, face photo or video) without the additional information strictly kept confidential by us within the KUH Information System. For the General Data Protection Regulation (GDPR)-compliant pseudonymization [23] we excluded from the timelines, CT/MRI slice panels, tables, and texts the following individual or individualizing data: name, gender, date, month and year of aSAH, clock times, and time period lengths (except the time to death in Fig. 5B). The Kuopio IA Database does not contain face photos, and the 45 DC patients were not media personalities. Concerning (i) hostile hacking or (ii) legal hacking resistance testing, the hackers must breach the KUH Information System (immediate alarm), manage to enter various databases, and then cross-link an immense amount Finnish text and imaging data to obtain the correct personal identity code of any of the 45 DC patients. The CT/MRI slices, three or four per patient (Fig. 5), do not allow the face recognition, e.g., among the facial photos or videos in the social media. In contrast, providing the entire packs of CT or MRI slices would allow 3D face segmentation, so much so that “de-facing” algorithms have been presented [88, 101, 111].

### Performance of EMS and KUH logistics until the sIA occlusion

The present 45 DC patients, 11% of the 402 H&H 3–5 patients, required swift logistics in the spread-out population served solely by KUH (Fig. 1). The mutually agreed and honed logistics with CT teleconsultation since 2004 functioned satisfactorily, also in weekdays and duty hours (Table 3) [28, 105], with a few considerations.

The 112 call dispatchers’ guidance of the ambulances seemed acceptable, with a few lengthy arrivals (Table 2) [8]. The paramedics did not intubate but could install supraglottic airway devices. In a few cases, the times to intubation in relation to GCS on ambulance arrival were lengthy (Fig. 4) [30, 46, 54, 94, 95]. The ambulances did not carry CT scanners [31, 107], considering direct transfer to KUH in case of SAH after teleconsultation. Helicopters with EMS physicians to take over after the paramedic contacts were considered case-by-case [72]. The EMS charts were hand-written, but now a national electronic platform for prehospital emergency care is being adopted [74].

### Re-bleeds since the ictus until the sIA occlusion

In a Norwegian cohort of 486 aSAH patients, with the EMS logistics of the catchment area, 9.7% had re-bleeds prior to aneurysm repair, and the frequency of re-bleeds increased from H&H grades 1 to 5 [94, 95]. Among our 45 DC patients with H&H 3–5 at KUH arrival, a total of 29 (64%) patients had suspected or CT-verified re-bleed(s) until the sIA occlusion (Figs. 2 and 4, Tables 1 and 4). In studying the 29 timelines (Fig. 4) and the EMS and KUH logistics (Table 3), it is difficult to tell whether more haste would have prevented re-bleeds. From the first medical contacts, there were 31 re-bleeds or one per 22 h (Fig. 4) of the total exposure time of 671 h until the sIA occlusion. Tranexamic acid was started after the first CT. Tranexamic acid seems to reduce re-bleeds, but paradoxically, the clinical outcome would remain unchanged [75]. Significant re-bleeds may also occur during sIA clipping or endovascular occlusion [45, 115].

### Re-bleeds and true incidence of aSAH in defined populations

Of our 45 DC patients, 13 (29%) had *suspected or clinical re-bleeds before the first medical contact* (Fig. 4, Table 4). In a Norwegian cohort, a second thunderclap headache with or without loss of consciousness and a sudden deterioration in GCS were considered a *clinical re-bleed* [94–96]. “Minor leak” just means a missed diagnosis of aSAH [68, 113]. Re-bleeds may conceal the real aSAH incidence in defined populations. Among sudden deaths outside hospital, acute aSAH is one potential cause, verifiable at autopsy [55], possibly with adjunct cadaveric CT and CT infusion angiography [40]. At autopsies, however, it may be difficult to distinct one bleed from two bleeds. Re-bleeds may worsen the patients’ clinical condition and prognosis so profoundly that they are (i) not transferred to the neurointensive care and thereby (ii) not entered into clinical aSAH databases [81].

### aICH, perihematomal edema, brain ischemia, and final brain injury areas

In our DC cohort, aICHs not just (i) teared hematoma cavities within the brain tissue, later to be filled with cerebrospinal fluid (CSF), but also provoked adjacent (ii) areas of perihematomal edema [11, 34, 76] and (iii) subsequent brain injury areas [18], several of them massive. The aICHs at the MCA bifurcation proved particularly noxious for the brain [26, 93]. Of the 27 clippings with aICH, 18 (67%) started within eight hours of the 112 call, the goal time window in the present Dutch ICH Surgery Trial (NCT03608423) [91]. Intraoperative CT [35], not available, would have disclosed possible residuals. Endovascular occlusion did not spare from brain infarcts adjacent to aICHs.

*Primary causes* of ischemia include the tearing of small vessels by the burst of arterial blood, locally increased pressure, and brain herniation. *Secondary causes* of brain injury [79] include neuroinflammation [24, 44], brain edema [116], microglial activation [10], leukocyte infiltration [57, 117], perihematomal edema [76], impaired glymphatic drainage [2], delayed brain edema resolution [80, 86], delayed blood clearance [71, 73], and microemboli [12, 79]. *Iatrogenic causes* include manipulation, temporary artery occlusion, and accidental branch or perforator occlusion during sIA occlusions.

## Strengths and limitations of the pilot study

We may be the first to illustrate *all patients’* individual timeline and serial imaging panels from the EMS contact to the outcome at 12 months in any acute brain insult cohort. The strengths derive from the tax-paid Finnish health care system and the automatic archival of clinical data, using the Finnish identity codes, in the national registries. Finland is divided into exclusive catchment areas between the five university hospitals which results in cohorts that are minimally selected and biased. The Kuopio Database contains all aSAH patients admitted from Eastern Finland (Fig. 1) and allows to reconstruct their clinical timelines, including data in other hospitals and national registries [5, 100].

There are also limitations. Our pilot study is retrospective while the database was prospective in the study period. We chose secondary DC as an indicator of escalating aSAH, so the 45 patients represent only 6% of the entire aSAH study population. In retrospect, there was shortage of perfusion and MRI studies. Space did not allow to discuss in detail various aspects of aSAH along the timelines. The panels here may at the present be hard to compile in other neuroacutology services.

Of the 20 DC survivors, there were 15 CT scans and five MRI scans available at about one year (Fig. 5B). They reflected (i) the lost brain areas caused by aICH and (ii) injured brain areas. It is challenging to compare the two modalities for the final brain outcome; naturally, a much larger number of MRI scans would be preferable.

## Suggested development—individual timeline and serial imaging panels to fill present knowledge gaps

1. Clinical researchers and journals of acute brain insults could consider the publication—*with strict and shared pseudonymization practices*—of the patients’ individual (i) timeline panels from the EMS contact and (ii) serial brain imaging panels until at least 12 months. This approach would support Personalized Acute Care. Any findings associated with *the final brain outcome* could be supported with the serial imaging panels [109, 118]. Such illustrative panels—with basic animation—require user-friendly interfaces with timeline construction tools.
2. Acute brain insult databases with individual timelines and serial imaging could create national and international registries with constant accrual from the allied institutes, including digital radiology, histology and genomics, and biobanking of tissue specimen. Such registries may allow in silico and *virtual randomized clinical trials* (*RCTs*) [62] in comparison to more laborious RCTs, as well as re-analysis of pseudonymized “raw data.”
3. Machine learning (ML) reading of acute and serial neuroimaging (CT CTA MR MRA perfusion) in daily clinical use could depend on such databases and registries as *ML teaching resources*, including outcome predictions [17, 60].
4. The IT systems of the hospital catchment area and allied stakeholders should provide individual timelines (in minute scale) from multiple data sources, since the EMS contact through the (neuro)intensive care until the final outcome. At EMS phase, the patient’s timeline should *proceed near real time* on the monitoring screens.
5. *Any branch of acute or elective Personalized Care* will benefit from the individual timelining and serial imaging, e.g., in daily practice, follow-up, *quality control for timely and equal access to clinical services*, and cost analysis. Such transparent analyses of data may illustrate information, mechanisms, and causal relations, possibly overlooked or not presented before. With tens, hundreds, or thousands of individual timelines on the screen, new tools for *visual dissective and comparative multivariate analysis* are required.

## Suggested further research and development—aneurysmal SAH

Today, clinical research on aSAH is hampered by generalizations and inexact scales of volume, causality, and kinetics of events. For example, “vasospasm” is jargon for delayed ischemic injury, simplifying pathogenesis and potentially misguiding scientific understanding [6, 12, 79, 112]. Grading of subarachnoid blood with the modified Fisher scale [61] fails to specify the aICH and aIVH “clots.” With the present approach, the analysis of individual blood distribution and 3D angiographies to detect which main trunks and segments really become “vasospastic’” in the patient’s timeline and how that correlate in (i) perfusion studies and (ii) final brain outcome [3, 16, 90] in the machine learning (ML) analysis becomes feasible.

The contents of (i) early ischemic brain areas and (ii) perihematomal edema areas could be studied in detail with ex vivo MRI combined with histopathology [36, 103] in aSAH patients who succumbed during their neurointensive care. It remains to be seen how salvable these “penumbral” areas become with novel therapies [32, 53, 77, 96].

To reduce neuroinflammation—within reason—blood burden [70, 93] could be promptly (i) released from aICH cavities and (ii) rinsed with catheters from cisterns [25] and ventricles [43, 49, 85, 102]. Continuous infusion of novel drugs into main cerebral arteries, cisterns, and ventricles is conceivable [43, 63].

## Clinical conclusions

In our pilot study, the pseudonymized timeline panels and serial brain imaging panels, indicating the patients by numbers, allowed the presentation and comparison of individual clinical courses. An obvious application would be the quality control in acute or elective medicine for timely and equal access to clinical care, in support of Personalized Medicine.

## Consent, data, material, and/or code availability

All research consents, data, material, and coding are available for corresponding author.

## Data Availability

Major parts of our data are derived from Finnish national health registries, which are regulated by the Finnish Institute of Health and Welfare. By their strict instructions, we are not allowed to share our datasets openly in any form due to privacy requirements of Finnish law. Interested researchers can be granted permission to our datasets by individual evaluation and the final permission will be granted by Institute of Health and Welfare.

## Abbreviations

ACA: Anterior cerebral artery
ACo: Anterior communicating artery
aICH: Aneurysmal intracerebral hemorrhage
aIVH: Aneurysmal intraventricular hemorrhage
aSAH: Aneurysmal subarachnoid hemorrhage
aSDH: Acute subdural hemorrhage from aSAH
CH: Central Hospital
clip: Microsurgical occlusion
clipped: Microsurgical occlusion
CNS: Central nervous system
coil: Endovascular occlusion
coiled: Endovascular occlusion
CSF: Cerebrospinal fluid
CT: Computed tomography
CTA: CT angiography
dBI: Delayed brain injury
DC: Decompressive craniectomy
DSA: Digital subtraction angiography
eICH: Evacuation of aICH
EMS: Emergency medical services
eSDH: Evacuation of aSDH
EVD: Extraventricular drainage
GCS: Glasgow Coma Scale
GDPR: General Data Protection Regulation
GPS: Global Positioning System
H&H: Hunt & Hess scale
HEMS: Helicopter emergency medical services
IA: Intracranial aneurysm
ICA: Internal carotid artery trunk and bifurcation
ICG: Indocyanine green
ICH: Intracerebral hemorrhage
ICP: Intracranial pressure
ICU: Intensive care unit
IQR: 25% And 75% range
IVH: Intraventricular hemorrhage
Jo CH: Joensuu Central Hospital
Jy CH: Jyväskylä Central Hospital
km: Kilometer
KUH: Kuopio University Hospital
M1: M1 segment of the middle cerebral artery
Mbif: Middle cerebral artery bifurcation
Mi CH: Mikkeli Central Hospital
ML: Machine learning
MRI: Magnetic resonance imaging
mRS: Modified Rankin Scale
n.a.: Not applicable
n.r.: Not relevant
n.s.: Not significant
NICU: Neurointensive Care Unit
PACS: Digital image archive
PCo: Posterior communicating artery
RCT: Randomized clinical trial
Sa CH: Savonlinna Central Hospital
SAH: Subarachnoid hemorrhage
Sh: Shunt
sIA: Saccular intracranial aneurysm
TBI: Traumatic brain injury
